# A review of attitudes towards the reuse of health data among people in the European Union: The primacy of purpose and the common good

**DOI:** 10.1016/j.healthpol.2019.03.012

**Published:** 2019-06

**Authors:** Lea L. Skovgaard, Sarah Wadmann, Klaus Hoeyer

**Affiliations:** a Department of Public Health, University of Copenhagen, Øster Farigmagsgade 5, 1014 Copenhagen K, Denmark; b The Danish Center for Social Science Research, VIVE, Herluf Trolles Gade 11. 1052, Copenhagen K, Denmark

**Keywords:** Health data, Informed consent, Public attitudes, Review

## Abstract

Health data are used for still more purposes, and policies are enacted to facilitate data reuse within the European Union. This literature synthesis explores attitudes among people living in the European Union towards the use of health data for purposes other than treatment. Our findings indicate that while a majority hold positive attitudes towards the use of health data for multiple purposes, the positive attitudes are typically conditional on the expectation that data will be used to further the common good. Concerns evolve around the commercialisation of data, data security and the use of data against the interests of the people providing the data. Studies of these issues are limited geographically as well as in scope. We therefore identify a need for cross-national exploration of attitudes among people living in the European Union to inform future policies in health data governance.

## Highlights

• Studies of attitudes towards reuse of health data mainly from the UK.
• Studies show lack of awareness of current usages of health data among people living in the EU.
• Studies report positive attitudes towards the sharing of health data.
• Concerns about commercial use of health data is expressed in the studies.
• Attitudes towards informed consent are inconsistent.

## 1 Introduction

Personal health data are collected from patients on an unprecedented scale [1]. Data are used for a multitude of purposes, including research, planning, quality assurance and police work [2]. In some cases, data availability is also used by national governments to attract international investments [3,4]. Health data are often described as a “goldmine” with vast possibilities [[5], [6], [7]] and the European Commission has described health data as a unique resource due to the possibility of doing prospective as well as retrospective research at low costs [8]. OECD similarly encourages member states to develop and implement health data governance frameworks that secure privacy while also enabling the reuse of health data [1,9,10]. Correspondingly, the European Commission call on member states to invest in digital transformation of their health services serving the double aim of improving population health and strengthening the digital single market in Europe [11]. The European Council promotes adaptation of e-Health infrastructures to facilitate accumulation, exchange and use of health data [12] and the EU also promotes the FAIR principles to further enhance the reuse of research data by making them Findable, Accessible, Interoperable and Reusable [13]. With the adoption of the General Data Protection Regulation (GDPR), the European Union (EU) has shown its commitment to promote data exchange within and between member countries while also increasing data protection [14]. In short, policymakers across Europe are determined to ensure better access to and increased use of health data for treatment as well as other purposes.

Meanwhile, cases have developed in some European countries demonstrating that the reuse of health data is a sensitive matter that can develop into a publicly contested issue. In England, the collection of data in the *care.data* scheme, where NHS Digital collected health data from general practice to use for research and planning by actors within and outside NHS, caused a public controversy [10,15]. In Denmark, a similar case, where health data collected from general practice by a quality appraisal unit was reused for health research and administrative purposes, caused public reactions [16]. In both cases, the legality of the databases and purposes of data reuse were questioned in the public debate, which lead some patients to request that their data were deleted. If public debate causes some people to ask for withholding of health data, this indicates a need to understand better under which conditions the use of health data is seen as acceptable by people in the EU. This is important to ensure that governance is aligned with the views of people in the EU member states - but also to ensure the validity of the data recorded.

Previous reviews have suggested that people generally hold a positive attitude towards the reuse of health data [17,18], but also pointed to a lack of awareness about practices and patient rights in relation to the sharing of health data [18]. Nevertheless, concerns about privacy, confidentiality and data security have also been reported [17,18]. These reviews have been restricted either to studies using qualitative methods and have focused solely on the reuse of health data for research purposes [17], or to attitudes toward a specific data governance aspects such as informed consent [18]. With this review, we update the understanding of public attitudes towards the reuse of health data focusing on the EU. We impose no methodological restrictions and include the reuse of data for all purposes as well as all aspects of public opinion. Hence, the aim is *to explore expressions of attitudes among people living in the EU towards the use of health data for purposes other than treatment.*

Studies of attitudes often presuppose ideas about a uniform and bounded group referred to as “the public”. It is, however, often unclear who counts as members of this “public”. When studying attitudes a selection is taking place, e.g. based on assumptions about mental capability and age range, but these assumptions often remain implicit. Furthermore, these choices can have a political character, for instance there are political disagreements about whether unregistered migrants should be counted as part of the public in a given country. In this way, the methodology of a study constructs its own public. In this article, we refer to *people living in the EU* without imagining a bounded and exhaustive “public” and to avoid the impression of a uniform “public”. We refer to *health data* as data collected in relation to clinical care or other routine contacts to the healthcare sector, and understand the *reuse of health data* as use for purposes beyond clinical care [19]. We understand *views* and *attitudes* as a normative predisposition arrived at in the course of either qualitative inquiry or surveys that may, but need not, inform future action. They are nevertheless important indicators of legitimacy. Attitudes are context dependent, and different methodologies will allow people to arrive at different positions.

## 2 Methods

### 2.1 Search strategy

Studies were identified through searches in the electronic databases Embase, PubMed, PsycInfo and Sociological Abstracts during 23^rd^–26^th^ of August 2016 using free text searches and the indexation system available in each database. The search was updated in January 2019. In addition, reference lists of obtained literature were reviewed and citation searches undertaken for the included studies (Web of Science). The search model can be seen in Table 1.

**Table 1:** Search Model.

Step 1: (lay perspective*) OR (lay view*) OR (lay attitude*) OR (lay opinion*) OR (lay preference*) OR (public perspective*) OR (public view*) OR (public attitude*) OR (public opinion*) OR (public preference*) OR (patient perspective*) OR (patient view*) OR (patient attitude*) OR (patient opinion*) OR (patient preference*) AND Step 2: (health data) OR (health record*) OR (electronic health record*) OR (patient record*) OR (medical record*).

### 2.2 In- and exclusion criteria

**Fig. 1:**
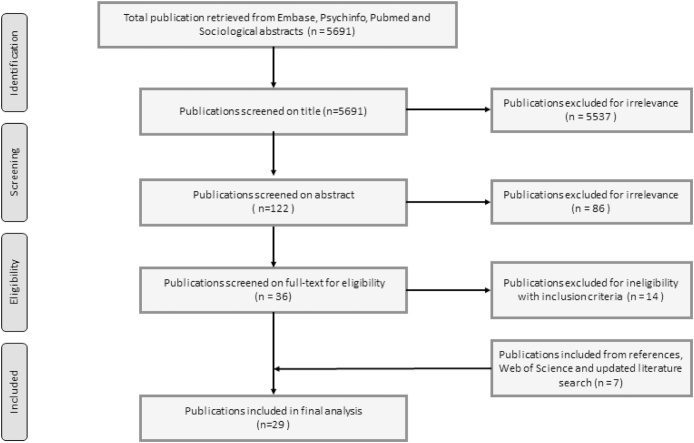
In- and exclusion of studies.

Fig. 1 presents a flow diagram of the in- and exclusion of studies. Due to a technical challenge in the initial data search duplicates were not removed *before* the initial screening of titles, but during the screening of titles. However, this should have no practical effect on the included studies.

[Author] screened all titles and abstracts for relevance. In cases of doubt, [Author and Author] were consulted. The in- and exclusion of studies followed the criteria listed in Table 2.

**Table 2:** In- and exclusion criteria.

Criteria	Description
*Topic*	Studies that did not concern the views of people living in the EU on reuse of health data for purposes other than treatment were excluded.
*Date*	Studies published after 2000 were included.
*Type of data*	Previous reviews have focused on attitudes towards the use of biological data [21,22]. This review focuses on attitudes towards the use of health data collected in conjunction with clinical care.
*Peer review*	Only peer-reviewed literature was included to avoid inserting a bias as a result of grey literature being primarily published in local languages not accessible to the authors.
*Geography*	Studies from EU member states were included. Studies from the UK were included based on the expectation that some exchange of health data between EU member states and the UK will continue despite UK leaving the union.
*Language*	Studies that were accessible in English, German or the Scandinavian languages were included.
*Duplicates*	In cases where several studies were conducted on the same dataset, only one study was included, unless the studies reported on different parts of the dataset or new analytical approaches were used.
*Research participation*	Studies exploring patients’ views on the linkage of medical health records to data generated in a clinical trial they participated in were excluded as these patients had already once agreed to their data being used for other purposes than treatment.
*Study design*	Reviews were not included but used to identify further primary research.

All studies underwent quality appraisal based on four basic questions (Appendix 1 in Supplementary material) inspired by Dixon-Woods et al. (2006) [23]. Due to the exploratory nature of this review, the quality appraisal concerned basic levels of quality, intern validity and relevance of the studies rather than a detailed evaluation of potential biases. No studies were excluded due to poor quality.

### 2.3 Data analysis

The review was undertaken as a configurative literature synthesis [24,25]. The included studies were coded for thematic content using the software Nvivo. The coding followed an inductive approach using line-by-line coding to let the themes emerge based on the content of the texts [26]. Each study was read by at least two authors and coded by one author based on discussion with the co-authors. Themes were discussed until agreement was reached.

## 3 Results

Twenty-nine studies were included (Fig. 1). An overview of the articles, including author information, setting, methods, study population and aims of the study, can be found in Table 3. The majority of studies explored attitudes towards the use of health data for *research* [17,18,[27], [28], [29], [30], [31], [32], [33], [34], [35], [36], [37], [38], [39], [40], [41], [42], [43], [44]]. Most studies asked for views regarding research in general [17,18,[27], [28], [29], [30], [31], [32], [33], [34], [35], [36], [37], [38],^44^], while six studies focused on *specific* registries [[39], [40], [41], [42], [43],^45^]. Eight studies explored views on the use of data for purposes other than treatment and research, namely the *evaluation of a screening programme* [46], *planning and polic*y purposes [32,34,35,44], the production of *performance reports* [47] and quality assurance (clinical *audit*) [48]. One study explored whom the participants would be willing to grant access to their health data [49] and one study investigated concerns regarding access for non-medical personnel and private companies [50]. The vast majority of studies were conducted in the UK [17,18,27,31,[33], [34], [35], [36], [37],[39], [40], [41], [42], [43], [44], [45],^47^,^48^,[51], [52], [53]]. Six studies from other countries were identified, including Italy, Germany, Finland, Netherlands and Ireland [[28], [29], [30],^38^,^46^,^49^] and one study was a pan-European survey [50].

**Table 3:** Overview of studies included in the review.

### 3.1 Low awareness

Generally, the studies reported low levels of awareness among the respondents about the reuse of health data. Studies found limited awareness of specific disease registries [30,40], the existence of a database from general practice [44], the content of the electronic health record (EHR) [28,48], anonymization practices [43], data sharing practices [17,31,37,48,49], the types of data used for research and the types of research conducted [17]. Only two studies reported general awareness among the informants about the existence and content of the EHR [29,35]. The lack of awareness is an important finding in itself and should be kept in mind, when other results are assessed.

### 3.2 Positive attitudes conditioned on the perception that data use serves the common good

The studies included in this review generally found positive attitudes among respondents towards the use of health data for research purposes [[30], [31], [32],^41^,^44^,^45^,^53^], the evaluation of a screening programme [46], quality assurance [48,50] and for planning and policy purposes [32,34,44]. Importantly, the positive attitudes seem to be conditional on the understanding that the use of health data will further the common good [17,31,39,41,42,52,53], for example through a better understanding of diseases [29,34,37], improvement of treatments [34,53] or more efficient health care planning and delivery [34,40].

### 3.3 Unacceptable forms of data reuse

Health data reuse was seen as unacceptable when: 1) it was perceived not to serve the common good, and 2) it was seen as potentially conflicting with the interests of patients providing the data. Concerning the first, a common scepticism was identified in relation to the commercialisation of health data [[42], [43], [44],^49^,^52^,^53^], either in the form of private companies profiting from data [17,42,49,53] or via the sale of health data to private companies [42,52]. Some respondents, however, found the sale of health data acceptable, as long as money is fed back into the public health care services [36,52] and thereby used to further the common good. Also, pharmaceutical companies having access to health data was seen as positive by some [17,31,41] and negative by others [17,36,39,41,50,53], depending on whom the respondents believed would benefit from the usage. Regarding the use of data to the disadvantage of patients, concern was expressed about employers having access to health data [36,39] due to fear of negative implications for employment [29,52]. Furthermore, aversion towards the sharing of health data with insurance companies was expressed in several studies [31,33,34,36,37,41,44,45,[48], [49], [50],^52^] because of fears that data could be used to decline insurance claims or increase premiums [29,31,41,52]. Aversion towards health data being used for marketing purposes was also expressed [39,52]. One study reported scepticism among patients about the use of health data for the publishing of performance information, because they found it to be of limited use for their choice of provider [47]. A minority appeared opposed to the use of health data irrespective of the purpose [29,33]. In one study, a minority even expressed opposition to let their own general practitioner (GP) view their general practice medical records [33]. In another study, some patients stated that they would only want to share *anonymous* data with their GP [29].

### 3.4 Sensitive data

Certain types of data appeared to be particularly sensitive for some people to share. The perceived sensitivity of data seems to depend on whether the information was seen as potentially stigmatising or the disclosure of data could have any other adverse effects for the patients. Data on alcohol or substance use [21], mental health (e.g. personal problems, depression, anxiety) [29,36,37,41,51,52] or sexual health (e.g. lack of libido, erectile dysfunction, contraception) [29,36,41,51,52] appeared to be particularly sensitive. One study also reported aversion among some persons towards the sharing of information about financial and social issues (e.g. about life insurance and being laid off work) in a national database [51].

### 3.5 Data security and data management

Data security and data management practices were recurrent themes in the studies. Some respondents expressed faith in data security [17,37,39], but a majority of the studies found that people generally worried about the security of data and feared data leakage [29,31,[34], [35], [36],^42^,^44^,^52^]. Doubts were expressed about the competencies and routines of those handling the data [17,42], including the adherence to guidelines for data management and storage of data [17,29,31]. Though the effect of anonymization or pseudonymization was questioned by some [31,42,43], respondents generally expressed a preference for data to be shared anonymously for research, audit and policy-planning purposes [32,36,45,48]. Some people, however, doubted whether data were anonymous and handled appropriately, and feared that privacy or confidentiality could be breached [37,42,43,52].

### 3.6 Requests for information about data reuse

A desire to be better informed about which data were extracted and for which purposes was also expressed in several studies [17,30,34,41,45]. In two studies, some respondents expressed the view that information about the use of health data was unnecessary or even burdensome [31,37]. Those who requested information gave various reasons for this, including the hope that it could be beneficial for their own treatment [31], that they wanted to be able to make informed decisions about research participation [36,41,52,53], to avoid anxiety based on misconceptions of data use [37] or because giving information was seen as “common courtesy” [36,52].

### 3.7 Attitudes towards informed consent

The majority of studies explored attitudes towards informed consent [[27], [28], [29], [30], [31],^33^,^35^,^37^,^38^,^39^,[41], [42], [43],^45^,^46^,^52^,^53^]. Some studies sought to reveal preferences for different consent models [28,30,37,39,[41], [42], [43],^45^,^52^]. Others explored whether people wanted informed consent [27,33,38] and whether the anonymization of data affected the preference for this [29,31,35,37,46,53]. Table 4 presents an overview of different consent models presented in the studies, along with stated attitudes towards the models. The models are not all mutually exclusive, but represent the choices offered in the respective studies.

**Table 4:** Seven consent models found in the literature.

Consent model	Attitudes toward consent model
Explicit consent *Consent is sought every time data are used*	13.4% preferred this model out of four possible models (n = 423) [30]. The least preferred of four possible consent models (n = 28) [52].
Dynamic consent *Patients can choose with whom data will be shared. Patients can change preferences at any given time.*	Patients appreciated the sense of control given by dynamic consent (n = 40) [37].
Individual consent *Patients can choose different levels of involvement (e.g. in anonymous descriptive studies, clinical or non-clinical research)*	Patients found that being able to choose levels of involvement would encourage participation (n = 68) [39].
Meta consent *1. One consent for one field of research* *2. One consent for one research registry*	1. 44.6% of respondents preferred this model out of four possible models (n = 423) [30]. 2. 41.3% of respondents preferred this model out of four possible models (n = 423) [30].
Consent for Contact *Patients sign up for a registry and allow researchers to contact registered individuals if they meet the criteria for a specific project*	The frequency with which participants would be contacted seemed to be of importance for attitude, though no acceptable frequency was agreed upon (n = 37) [41].
Consent agreement with GP *GPs act as ‘gate-keepers’ for researchers’ access to patients’ health records. Consent is given once to the GP.*	83.7% of respondents would be willing to let their GP decide when to provide anonymous information to researchers (n = 1575) [28].
Opt out *Inclusion as default. Patients have to actively let the relevant authority know if they do not want health data to be used.*	Patients expressed dissatisfaction with this model (3537 tweets from 904 twitter accounts) [43], (201 entries from 171 bloggers Blog entries from 85 individuals on two blogs from The Guardian) [42]. The most preferred model out of four possible (n = 28) [52]

For the use of health data for research, the share of respondents who stated that consent should be sought before data are used varied between 12% [27] and 56% [38]. In the Finnish study, respondents were almost equally split between wanting consent to be sought “every time”, “sometimes” and “never” for research purposes [30]. If consent for the use of health data was to be legally required, only 13.4% stated that they would prefer explicit consent, while the rest would prefer some form of meta-consent (cf. Table 4) [30]. In another study 10–11% of respondents wanted to be asked, indicating a wish for explicit consent, if data was used to inform audit and the publishing of performance information [48]. For teaching purposes, the percentage varied between 10% [27] and 44.3% [33]. Though one study made a distinction between consent being sought always and in some cases only [30], none of the studies informs us how often people think consent should be sought.

A majority of respondents stated that they would allow the use of health data for research and quality assurance purposes without consent, as long as data were *anonymous* [31,37,48,53], while a minority appears to accept the use of *identifiable* health data also without consent [48]. In one qualitative study, participants expressed preference for consent to be sought every time, if *anonymous* or *identifiable* data were used for research [29]. When *identifiable* data were used to evaluate a screening programme, the vast majority also preferred consent to be sought [46]. Some studies have explicitly investigated possible differences in patients’ preference for consent, depending on whether data are anonymous or person identifiable, but they report contradicting attitudes [33,35].

## 4 Discussion

In this review, we have explored attitudes among people living in the EU towards the reuse of health data. Only a few studies conducted outside Britain was identified. This stresses a need for further empirical studies beyond the British context to be able to assess differences and similarities across various constituencies. A transnational study among EU member states, which include both qualitative and quantitative methods, would secure accessibility of information on opinions of people living in the EU about reuse of health data beyond the local context.

Across the reviewed studies, it was striking that many respondents did not know which health data were being shared and how they were being used. Hence, when interpreting the findings of the studies, it is important to bear in mind that patients expressed more generally formed attitudes without knowing the specificities of the practices involved. The studies included in this review do not provide possible explanations for the low levels of awareness which warrants future studies to address this question. Many respondents in the studies expressed a wish to be better informed about the storage and reuse of health data. Considering the fact that people are asked to form an opinion about a topic that they have just stated limited knowledge of, it is perhaps not surprising that they request information.

Generally, the studies found that a majority of people hold positive attitudes towards the use of health data for purposes other than treatment. However, some forms of data use require support from more than a simple majority: for registry studies to yield valid results researchers have pointed out that inclusion rates should be at least 90% [46]. This raises a regulatory dilemma about how autonomy should be weighed against the ability to produce valid register research. In line with previous studies, we found that positive attitudes reported in the studies were often conditional on the understanding that data would be used to further the common good [17], and some respondents were opposed to the reuse of health data when they believed this not to be the case. Hence, the purposes of data use mattered to the respondents. For some people, privacy appeared to overrule all other concerns. Concerns expressed in the studies particularly related to the fear that data might be used to the disadvantage of patients (e.g. by insurance companies) and that data would be commercialised leading to private companies profiting from patients’ health data. It has previously been suggested that citizens in the EU view medical information as sensitive [54]. Our findings indicate that the perceived sensitivity of data seems to depend on whether disclosure of the information is viewed as stigmatising or potentially harmful to patients. Another common concern was that data would not be managed appropriately and that data security was insufficient to prevent data leakage or inappropriate access. These concerns stress the need for policy makers to address issues relating to data management and data security as it has also been pointed out by the EU (GDPR) and OECD [10,14]. Regarding attitudes towards informed consent, no clear picture of the legitimacy of various consent models emerged, despite this issue being the focus of most studies. Given the vast attention on informed consent it would be relevant for future studies to map existing consent procedures across EU. It is worth noting that informed consent did not appear to be an issue raised spontaneously by the respondents in the studies. Rather, the issue was typically raised by the researchers who asked informants directly about whether they preferred consent and about their preferences for specific consent models. Hence, the focus on consent might reflect regulators and researchers’ interest in consent rather than mirror the participants’ concerns [55]. Our point is not to argue for or against informed consent but to draw attention to the fact that the issue takes up so much space in the studies that it leaves little room for discussion of other issues that are of clear importance to patients, such as conditions of commercialization, data security, and alternative forms of protection of patient interests. Considering the policy attentiveness to data protection and data security [10,14], and the concerns expressed in the included studies, future studies should address attitudes towards different ways of handling data security to inform future policies.

Some variance in expressed attitudes was identified among the studies. This may reflect cultural differences among the countries where the studies have been undertaken, but it may also reflect methodological differences. The response rate being below 50% in several studies [[28], [29], [30],^33^,^38^,^41^,^45^] also introduces a risk of selection bias. Depending on the study population, some studies may be expected to elicit more positive attitudes than others, for example when study participants are recruited from a patient involvement group [52]. Likewise, more negative attitudes may be expected in, for instance, studies of online reactions to controversial cases [42,43]. Contrary to our expectation, no systematic differences in opinions were found in the studies from the UK before and after the care.data case had unravelled. Concerning the risk that variance across the studies is due to methodological differences, in particular framing should be considered. For example, Campbell and colleagues (2007) asked people whether they would “prefer to give […] permission” for “doctors” to access their health data in order to “provide better information for the teaching of healthcare professionals” [27], whereas Ogden and colleagues (2005) asked patients whether “medical students” should be “allowed to see” their “medical record” and provided the options “never”, “only in emergency, or at patient’s discretion” (i.e. explicit consent) or “whenever the specified group wishes, or at the GP’s discretion” [33]. Answers to these questions are difficult to compare. The framing of the information respondents are provided with might also affect attitudes. Thus, the possible effect of the nature of information given, for example the effects of limiting the information to positive implications for research [52] or negative effects of consent [18], should be taken into account. Instead of viewing the variance as inconsistency it could also be a manifestation of the fact that no single and coherent public exists and that every public is always a construct in a given situation [56]. This calls for experiments into how different methodologies elicit different publics to understand better what we learn about public attitudes with different tools. Despite these limitations on comparability (and thereby accumulation of data from the different studies), we find it remarkable that similar concerns were expressed in relation to the commercialisation of data, data security and the use of data against the interests of the people providing the data.

Grey literature was not included in the review, which can be considered a limitation. However, it was excluded to avoid inserting a bias as a result of grey literature being primarily published in local languages not accessible to the authors.

## 5 Concluding remarks

Despite the general lack of awareness among respondents about the reuse of health data, some tendencies did appear across the studies. The findings of this review suggest that the use of health data for purposes other than treatment enjoys support among people living in EU asked in these studies, as long as the data are expected to further the common good. Purposes anticipated to conflict with this included the commercialisation of data and the use of data to potential disadvantage of patients. Concerned citizens do impact on the possibilities for using health data for purposes other than treatment, as became evident in the cases in Denmark and the UK, and the possible implications persist regardless of whether concerns are held by a representative part of the affected patients or not. Considering the scepticism of commercial use of health data identified across the studies, current European policies on making data available for private companies [3,4,13,[57], [58], [59], [60]] can involve a risk of public backlash. Finally, studies of these issues outside the UK are very limited, suggesting a need for a studies, both qualitative and quantitative, among EU member states as well as new methodological experiments comparing different tools for exploring attitudes in the same setting.

## Conflict of interest

The authors declare no conflict of interests.
