# Supplementary material for: A review of attitudes towards the reuse of health data among people in the European Union: The primacy of purpose and the common good
Source: Health Policy. 2019 Jun;123(6):564–71. doi: 10.1016/j.healthpol.2019.03.012 (PMC6558994; doi:10.1016/j.healthpol.2019.03.012)
Supplement: Supplementary file 1 [file mmc1.docx]

**Appendix 1**

Questions informing the quality appraisal

1. Are the aims and objectives of the research clearly stated?
2. Is the research design clearly specified and appropriate for the aims and objectives of the research?
3. Is it clear how data were collected, and is the data collection appropriate in relation to the aims of the research?
4. Is it clear how data were analysed, and does the data analysis appear to be sufficiently rigorous?
